# How competing risks affect the epidemiological relationship between vitamin D and prostate cancer incidence? A population‐based study

**DOI:** 10.1111/and.14410

**Published:** 2022-02-28

**Authors:** Ari Voutilainen, Jyrki K. Virtanen, Sari Hantunen, Tarja Nurmi, Petra Kokko, Tomi‐Pekka Tuomainen

**Affiliations:** ^1^ Institute of Public Health and Clinical Nutrition University of Eastern Finland Kuopio Finland

**Keywords:** cohort study, competing risk, incidence, prostate cancer, vitamin D

## Abstract

We hypothesized that controversial results regarding the epidemiological relationship between circulating 25‐hydroxyvitamin D, 25(OH)D, and risk of prostate cancer (PCA) incidence are partly due to competing risks. To test the hypothesis, we studied associations across 25(OH)D, PCA and death in 2578 middle‐aged men belonging to the Kuopio Ischaemic Heart Disease Risk Factor Study. The men were free of cancer at baseline, and the mean (SD) follow‐up time was 23.3 (9.1) years. During this period, 296 men had a PCA diagnosis, and 1448 men died without the PCA diagnosis. The absolute risk of developing PCA was highest in the highest 25(OH)D tertile (15%), whereas that of death was highest in the lowest 25(OH)D tertile (67%). A competing risk analysis showed that belonging to the highest 25(OH)D tertile increased the risk of PCA incidence and improved survival with the respective hazard ratios (HR) of 1.35 (95% CI = 1.07−1.70) and 0.79 (95% CI = 0.71−0.89). Adjusting for 10 covariates together with 25(OH)D did not significantly change the results, but the respective adjusted HRs for PCA and death were 1.20 and 0.87. To conclude, the competing risk analysis did not eliminate the direct relationship between 25(OH)D and PCA but rather strengthened it.

## 1 INTRODUCTION

Prostate cancer is the second most common cancer in men and the fifth most common cause of male cancer death (Sung et al., 2021). By and large, the risk of prostate cancer incidence is associated with older age, African ancestry and a family history but not with exogenous factors (Rebbeck, 2020). Moreover, many prospective studies have shown an association between adult height and prostate cancer incidence but based on Mendelian randomization analyses this association is indirect and linked to the family history of prostate cancer through heritability (Khankari et al., 2016).

Vitamin D deficiency is one of the most intensively studied possible exogenous risk factors of prostate cancer. So far, both cohort and case–control studies on the epidemiological relationship between vitamin D and prostate cancer incidence have shown conflicting results without clear explanations concerning the disagreements (Gandini et al., 2011; Gao et al., 2018; Gilbert et al., 2011). Anyway, there is a strong direct relationship between age and the risk of prostate cancer; the probability of developing prostate cancer is 40 times higher among men older than 70 compared with men younger than 50 (Siegel et al., 2019), and this relationship means that many men die for other reasons before they are old enough to develop prostate cancer. This for its part may explain conflicting results regarding the association between circulating 25‐hydroxyvitamin D, 25(OH)D, concentrations and the risk of prostate cancer incidence. As high 25(OH)D concentrations are associated with reduced mortality rates in prospective cohorts (Bouillon et al., 2019), prostate cancer incidence rates should be higher among men with higher 25(OH)D concentrations because they tend to live longer.

Overall, longitudinal and Mendelian randomization studies have found no evidence of an inverse relationship between low circulating vitamin D levels and prostate cancer incidence (Dimitrakopoulou et al., 2017; Heath et al., 2019; Travis et al., 2009; Yin et al., 2009), but some studies have reported a direct (Ahn et al., 2008; Albanes et al., 2011; Ong et al., 2021), a U‐shaped (Kristal et al., 2014), or even an inverted U‐shaped relationship (Brändstedt et al., 2012). One explanation for these contradictory results may relate to the vitamin D‐binding protein that modulates the impact of vitamin D status on prostate cancer risk (Weinstein et al., 2013).

Many cross‐sectional and retrospective studies associate vitamin D deficiency and insufficiency with prostate cancer mortality and, specifically, with aggressive prostate cancer (Choubey et al., 2017; Kumawat et al., 2021; Murphy et al., 2014; Özman et al., 2021; Xie et al., 2017). This association appears to be valid in middle‐income countries but not necessarily as evident in high‐income countries (Stanaland et al., 2017; Trump et al., 2009; Yaturu et al., 2012). Vitamin D deficiency and insufficiency also seem to predict prostate cancer mortality (Fang et al., 2011; Shui et al., 2012; Stroomberg et al., 2021), although it is difficult to distinguish the association of vitamin D status with prostate cancer mortality from that with all‐cause mortality (Fang et al., 2011; Stroomberg et al., 2021). Studies showing no interaction between circulating vitamin D levels and the prognosis of prostate cancer as recurrence‐free survival (Thederan et al., 2021) suggest that the association between vitamin D status and prostate cancer mortality mainly reflects the association between vitamin D and all‐cause mortality.

To sum, although the same men appear to be prone to both vitamin D deficiency and prostate cancer, there is not necessarily a causative relationship between the vitamin D status and prostate cancer. Circulating vitamin D concentrations and the vitamin D receptor, of which both are partly genetically determined, may be associated with the risk of lethal prostate cancer but most probably not with the risk of prostate cancer incidence (Guo et al., 2013; Shui et al., 2012; Torkko et al., 2020).

In this study, we hypothesized that the controversial results regarding the epidemiologic relationship between the vitamin D concentration in the blood and the risk of prostate cancer incidence can be partly due to competing risks, specifically, with respect to studies with long follow‐up periods. To test our hypothesis, we studied the association between circulating 25(OH)D concentrations and prostate cancer incidence in a large epidemiological data set and interpreted the results with respect to all‐cause mortality as a competing event.

## 2 MATERIALS AND METHODS

### 2.1 Study participants

The Kuopio Ischaemic Heart Disease Risk Factor Study (KIHD) is an ongoing population‐based follow‐up study (Salonen, 1988). Originally, the main purpose of the study was to investigate previously unestablished risk factors for acute myocardial infraction. Subsequently, the purpose has widened, and data include all causes of hospitalization and death. The causes of hospitalization are based on linkages to national health care registers maintained by the National Institute for Health and Welfare (permission number THL/93/5.05.00/2013) and the causes of death are based on linkages to registers maintained by Statistics Finland (permission number TK‐53‐1770‐16). Cancer cases are verified based on linkages to registers maintained by the Finnish Cancer Registry (permission number THL/93/5.05.00/2013). KIHD has received an ethical approval from the Research Ethics Committee of the University of Kuopio on December 1, 1983. In the 1980s, the committee did not necessarily provide reference numbers but identified studies by dates. All KIHD study participants have given written informed consent.

In this study, we used the main cohort of KIHD, which comprises a convenience sample of 2682 men, who lived in eastern Finland, in or near the city of Kuopio, and were middle‐aged, 42–60 years, at baseline between March 1984 and December 1989. We excluded participants who have had a cancer diagnosis before baseline (*n *= 51) as well as those with no 25(OH)D measurements at baseline (*n* = 53). Consequently, the final number of participants in this study was 2578. To estimate reliability of our results based on baseline 25(OH)D examinations, we repeated the competing risk analysis for 808 men who participated in the follow‐up 25(OH)D measurements and were still free of cancer between March 1998 and February 2001, 11 years after baseline.

### 2.2 Dependent and independent variables

In KIHD, cancer refers to malign neoplasms and carcinoma *in situ* excluding basal cell carcinoma and myelofibrosis based on cancer types by ICD‐10 (THL, 2011) and tumour behaviour by ICD‐O‐3 (WHO, 2013). This categorization follows the categorization used by the Finnish Cancer Registry. This study focussed on prostate cancer incidence referring to ICD‐10 code C61. We included all new prostate cancer cases diagnosed between baseline and 31 December 2018.

The main independent variable was the serum 25(OH)D concentration, precisely 25(OH)D_3_. At baseline, study participants gave blood samples. The laboratory of our institute separated serum and stored serum gel tubes mainly at −70°C, for a few years at −20°C. Starting in 2012, the laboratory determined serum 25(OH)D concentrations using a high‐performance liquid chromatography device (Shimadzu, Kyoto, Japan) equipped with a coulometric electrode array detector (CEAD, ESA). Coefficients of variation were less than 8% for controls with 25(OH)D_3_ from 27.5 to 88.0 nmol l^−1^. Participations in the Vitamin D External Quality Assessment Scheme four times per year served as the external quality control. Nurmi et al. (2013) describe the determination of serum 25(OH)D in detail.

Other independent baseline variables were smoking in pack‐years, alcohol consumption in drinks per week (in Finland, one drink refers to 12 g of pure alcohol), body mass index (BMI), inflammation status measured as high‐sensitive C‐reactive protein (hsCRP) in milligrams per litre, total physical activity (TPA) measured as metabolic equivalent hours (METh) per day and diet described as fibre and meat intakes in grams per day together with the total energy intake in kilocalories per day. According to the Cancer Society of Finland, these covariates comprise the biggest lifestyle risk factors for cancer (Pukkala et al., 2016). Moreover, we included age in years and height in centimetres as independent variables because these factors in general explain a great proportion of the prostate cancer risk (Rebbeck, 2020). To avoid overadjustment, we did not include fish intake, which is a major source of vitamin D, and sunlight exposure as independent variables. As a variable, the vitamin D concentration in the blood is in the potential causal pathway between the exposure (fish intake and sunlight exposure) and the outcome (prostate cancer). In KIHD, smoking status, alcohol consumption, TPA and diet are based on study participants’ own reporting (Lakka et al., 1994; Salonen et al., 1992; Virtanen et al., 2011; Voutilainen et al., 2001).

### 2.3 Statistical analyses

Since circulating 25(OH)D levels of people living in Scandinavia vary seasonally (Klingberg et al., 2015), we used season‐specific concentrations of 25(OH)D in the analyses. First, we identified two seasons, ‘summer’ and ‘winter’ that, respectively, represent 25(OH)D accumulated either during the sunniest or less sunnier months. Second, we distributed study participants into tertiles based on their season‐specific levels of 25(OH)D. To substitute missing values, we applied the mean imputation approach and used 25(OH)D tertile‐specific averages as substitutes. In many cases, mean imputation can even outperform multiple imputation, when imputing baseline covariates (Sullivan et al., 2018). The number of missing values per covariate ranged from zero (age) to 84 (pack‐years). In the analysis of baseline characteristics, we used the analysis of variance (ANOVA) and the Kruskal–Wallis test to detect differences across the 25(OH)D tertiles. IBM^®^ SPSS^®^ Statistics Version 27 served as a statistical platform for the baseline analysis.

To estimate the probability of prostate cancer incidence, we calculated the proportion of *a* to *b*, where *a* is the number of prostate cancer cases and *b* is the total number of study participants at risk.

To generate unadjusted survival estimates, we used the Kaplan–Meier model, and to generate hazards adjusted for additional covariates, we used the Cox proportional hazards model. In the models, numerical values from 1 to 3 represented the 25(OH)D tertiles in ascending order. In KIHD, the follow‐up time is based on linkages to national health registers. Regarding cancer, Finnish health care providers are obligated to report all diagnoses and their dates to the Finnish Cancer Registry and because KIHD is linked to it, the present study misses no cancer cases. Correspondingly, all non‐events were censored as they did not have the diagnosis in the registers. In this study, we applied the register‐based data updated till 31 December 2018.

In the competing risk analysis, we considered death as a competing risk and computed the competing risk regression (CRR) using the R version 3.5.3 (R Core Team, 2019) and the R package ‘cmprsk’ version 2.2.10 (Gray, 2020). The unadjusted regression included only 25(OH)D tertiles as covariates, whereas the adjusted regression included all baseline characteristics. The purpose of adjusting for several covariates was to estimate the magnitude of the effect of 25(OH)D on PCA incidence and death by comparing it to that of other potential risk and protective factors.

## 3 RESULTS

### 3.1 Baseline characteristics

Table 1 presents baseline characteristics of the 2578 study participants at different 25(OH)D tertiles. Briefly, distributions of covariates were skewed, and the total energy intake was the only characteristic that differed across the 25(OH)D tertiles based on both means and medians. The intake was highest in the first tertile (Table1). Overall, with respect to adequate 25(OH)D levels (NIH, 2021; Rosen, 2011), 68% of the study participants had the circulating 25(OH)D concentration <50 nmol l^−1^, and 7% of them had it ≥75 nmol l^−1^.

**TABLE 1:** Baseline characteristics of study participants distributed into groups according to season‐specific 25‐hydroxyvitamin D tertiles

Variable	1st tertile	2nd tertile	3rd tertile	*p*
*n*	859	860	859	N/A
25(OH)D (nmol l^−1^), ‘Summer’	38.1 (7.4)	56.8 (4.3)	78.4 (11.5)	N/A
	39 (15−49)	57 (49−64)	76 (64−120)	N/A
25(OH)D (nmol l^−1^), ‘Winter’	22.7 (4.7)	35.8 (3.9)	57.5 (12.9)	N/A
	24 (8−30)	35 (30−43)	54 (43−136)	N/A
Age (years)	52.9 (5.1)	53.0 (5.2)	53.2 (5.1)	0.422
	54 (42−61)	54 (42−61)	54 (42−61)	0.147
Height (cm)	173 (6.0)	173 (6.3)	173 (6.1)	0.419
	173 (152−189)	173 (150−194)	173 (129−191)	0.684
Smoking (pack‐years)	115 (127)	107 (128)	98.6 (112)	0.026
	80 (0−960)	68 (0−1162)	67 (0−800)	0.094
Alcohol (drinks per week)	6.3 (10.8)	6.5 (13.8)	6.0 (9.1)	0.616
	2 (0−112)	3 (0−238)	3 (0−122)	0.013
BMI (kg m^−2^)	26.9 (3.8)	26.9 (3.5)	26.9 (3.3)	0.990
	26 (17−49)	27 (19−45)	27 (19−40)	0.962
hsCRP (mg l^−1^)	2.7 (5.2)	2.3 (3.4)	2.3 (3.4)	0.025
	1.3 (0.1−89)	1.2 (0.1−38)	1.3 (0.1−45)	0.105
Physical activity (METh d^−1^)	40.2 (11.9)	40.2 (10.8)	40.5 (10.8)	0.825
	38 (4−90)	38 (12−92)	39 (16−89)	0.584
Fiber intake (g d^−1^)	25.4 (9.6)	25.0 (8.5)	24.9 (8.0)	0.450
	24 (5−99)	24 (5−78)	23 (6−68)	0.885
Meat intake (g d^−1^)	158 (81.6)	165 (86.5)	158 (81.6)	0.116
	147 (0−549)	152 (0−1079)	147 (0−703)	0.180
Total energy intake (kcal d^−1^)	2485 (660)	2423 (599)	2385 (586)	0.003
	2436 (527−6680)	2371 (954−5349)	2354 (825−4912)	0.012

Notes

Numbers indicate mean (SD) and median (range). The follow‐up refers to the period between baseline (1984−1989) and 31 December 2018. *p*‐value is for the between‐group difference.

### 3.2 Incidence rates of prostate cancer and all‐cause death

The mean (SD) follow‐up time was 23.3 (9.1) years. During this period, 296 men had a prostate cancer diagnosis, 1616 men died, and 54 of them died to prostate cancer. The mean (SD) age of prostate cancer diagnosis was 76.3 (9.2) years, and that of death was 74.2 (9.5) years. Men who died of prostate cancer had the prostate cancer diagnosis 5.2 years earlier on average.

**FIGURE 1:**
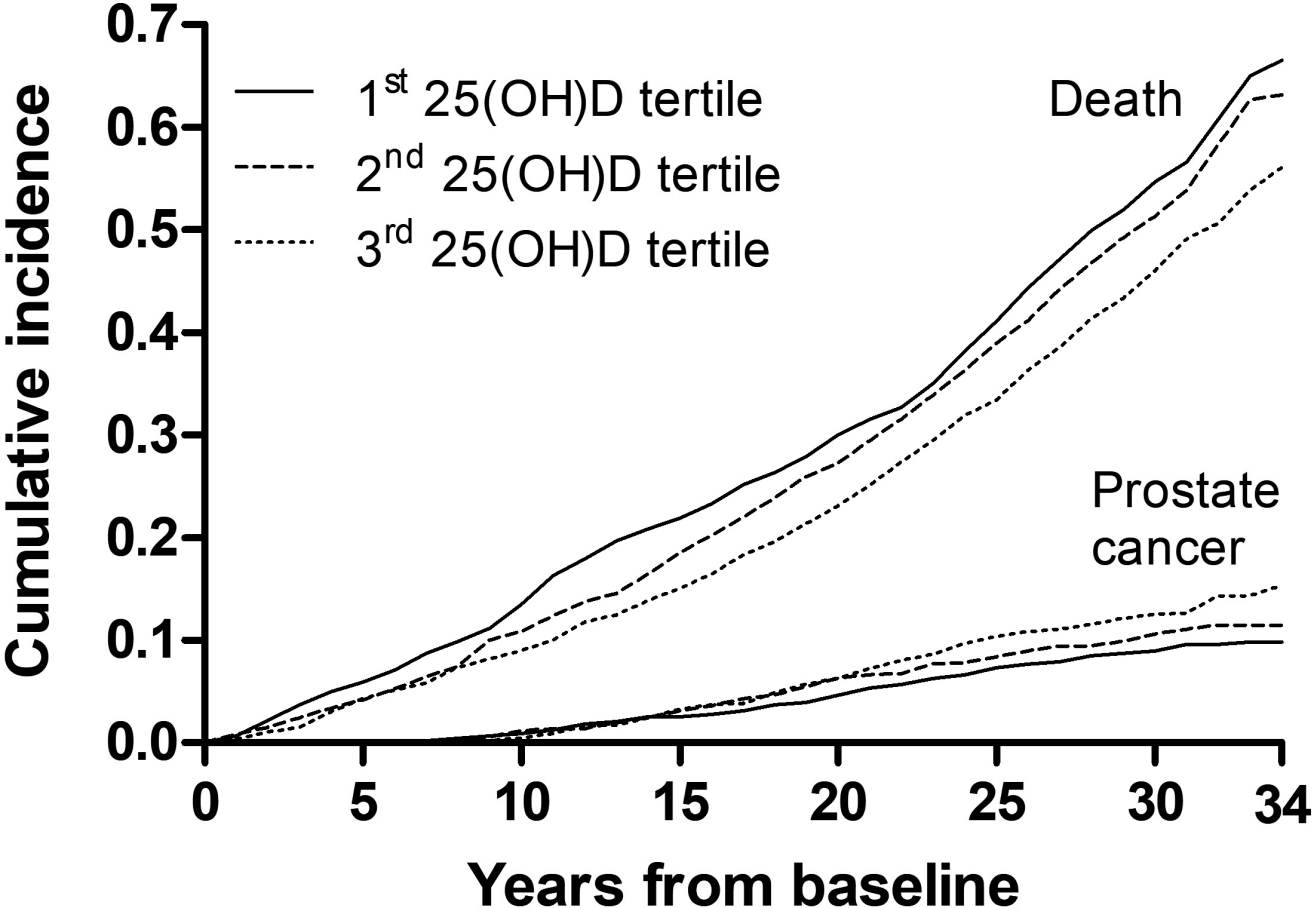
Cumulative incidences of all‐cause death excluding prostate cancer death and prostate cancer in 25‐hydroxyvitamin D tertiles based on a competing risk analysis

The probability of prostate cancer was 0.10 in the 1st 25(OH)D tertile, 0.11 in the 2nd tertile and 0.15 in the 3rd tertile. The probability of death, excluding prostate cancer death, was 0.67 in the 1st tertile, 0.63 in the 2nd tertile and 0.56 in the 3rd tertile. The probability of prostate cancer death was 0.02 in each tertile. Figure 1 illustrates cumulative incidences of prostate cancer and all‐cause death excluding prostate cancer death.

The probability of localized prostate cancer versus no prostate cancer (*n* = 150) was 0.05 in the 1st 25(OH)D tertile and 0.07 in the 2nd and 3rd tertiles. Of these 150 men, 19 (12.7%) died to prostate cancer by the end of 2018. A simple unadjusted Cox regression resulted in the following respective hazards ratios (HR) for localized prostate cancer in the 2nd and 3rd 25(OH)D tertiles compared with the 1st tertile: 1.50 (95% CI: 1.00−2.25, *p* = 0.052) and 1.33 (95% CI: 0.88−2.02, *p* = 0.181). Correspondingly, the probability of prostate cancer metastasized farther than to regional lymph nodes (*n *= 60) was 0.02 in the 1st and 2nd 25(OH)D tertiles and 0.04 in the 3rd tertile. Of these 60 men, 28 (46.7%) died to prostate cancer. The HRs for metastasized prostate cancer in the 2nd and 3rd 25(OH)D tertiles were as follows: 0.73 (95% CI: 0.37−1.45, *p* = 0.369) and 1.36 (95% CI: 0.75−2.44, *p *= 0.310). The tumour stage was unknown for 66 and non‐localized for 20 out of 296 PCA cases.

### 3.3 Competing risk analysis

For the competing risk analysis, the follow‐up endpoints were prostate cancer, death or the end of follow‐up period (31 December 2018). For 298 men, the endpoint was prostate cancer, of which for two men prostate cancer was a post‐mortem diagnosis, for 1448 death, and for 834 the date. The 1st 25(OH) tertile served as the reference category in the analysis, except for the unadjusted CRR that separately compared each 25(OH)D tertile to other tertiles.

Higher 25(OH)D concentrations improved the event‐free survival (EFS). In the unadjusted analysis, the hazard of an event, prostate cancer or death, decreased with the HR of 0.92 (95% CI: 0.87−0.98) across the 25(OH)D tertiles (*p* = 0.007). In the adjusted analysis, higher 25(OH)D concentrations still improved the EFS with the HR of 0.91, but age, smoking, alcohol drinking, higher BMI and higher hsCRP concentrations reduced it (Table 2).

**TABLE 2:** Results of the competing risk analysis

Covariate	EFS	Death CSH	PCA CSH	Death CRR	PCA CRR
25(OH)D per tertile	0.92**	0.93*	1.06*	0.79***	1.35*
	0.87−0.98	0.86−1.00	1.01−1.12	0.71−0.89	1.07−1.70
Adjusted for covariates	0.91**	0.87***	1.15	0.87***	1.20*
	0.86−0.97	0.82−0.93	1.00−1.32	0.82−0.93	1.04−1.38
Age per year	1.09***	1.11***	1.05***	1.09***	1.02
	1.08−1.11	1.09−1.12	1.02−1.07	1.08−1.11	1.00−1.04
Height per cm	1.00	1.00	1.01	0.99	1.01
	0.99−1.01	0.99−1.00	0.99−1.03	0.98−1.00	0.99−1.03
Smoking per 10 pack‐years	1.02***	1.02***	0.99	1.02***	0.98***
	1.02−1.02	1.02−1.03	0.98−1.00	1.02−1.03	0.96−0.99
Alcohol per drinks wk^−1^	1.01***	1.01***	1.01	1.01***	1.00
	1.01−1.02	1.01−1.02	1.00−1.02	1.01−1.02	0.99−1.01
BMI per kg m^−2^	1.03***	1.03***	1.01	1.03***	1.00
	1.01−1.04	1.02−1.05	0.97−1.04	1.02−1.05	0.97−1.03
hsCRP per mg l^−1^	1.03***	1.03***	0.99	1.03***	0.96
	1.02−1.03	1.02−1.04	0.95−1.03	1.02−1.04	0.92−1.00
Physical activity per METh d^−1^	1.00	1.00	1.00	1.00	1.00
	0.99−1.00	0.99−1.00	0.99−1.01	0.99−1.00	0.99−1.01
Fiber intake per g d^−1^	1.00	1.00	1.00	1.00	1.00
	0.99−1.00	0.99−1.00	0.98−1.02	0.99−1.00	0.98−1.02
Meat intake per 10 g d^−1^	1.00	1.00	1.00	1.00	1.00
	0.99−1.01	0.99−1.01	0.99−1.02	0.99−1.01	0.99−1.02
Energy intake per 100 kcal d^−1^	1.00	1.00	1.01	1.00	1.01
	0.99−1.01	0.99−1.01	0.99−1.03	0.99−1.01	0.99−1.04

Notes

Numbers indicate hazard ratios and 95% confidence intervals. Asterisks indicate statistical significance as follows: *p < 0.05, **p < 0.01 and ***p < 0.001.

The 1st 25(OH)D tertile served as the reference category, expect for the unadjusted CRR that compared the 3rd tertile to other tertiles.

Abbreviations: CRR, competing risk regression; CSH, cause‐specific hazard; EFS, event‐free survival; PCA, prostate cancer.

Higher 25(OH)D concentrations also decreased the cause‐specific hazard (CSH) of death with the HR of 0.93 (95% CI: 0.86−1.00, *p* = 0.039) but, simultaneously, increased the CSH of prostate cancer with the HR of 1.06 (95% CI: 1.01−1.12, *p* = 0.020). The adjusted CSH of death highlighted the same covariates as the adjusted EFS analysis, whereas age was the only covariate that statistically significantly associated with the CSH of prostate cancer (Table 2).

The unadjusted CRR concerning mortality resulted in the following HRs (95% CI) for the 25(OH)D tertiles, first to third: 1.20 (1.07−1.33, *p* = 0.001), 1.05 (0.94−1.17, *p* = 0.390) and 0.79 (0.71−0.89, *p* < 0.001). Regarding prostate cancer, the unadjusted CRR resulted in the following respective HRs (95% CI): 0.75 (0.58−0.97, *p* = 0.030), 0.96 (0.75−1.22, *p* = 0.740) and 1.35 (1.07−1.70, *p* = 0.012). In the adjusted CRR, the hazard of death decreased from low‐to‐moderate and from moderate‐to‐high 25(OH)D concentrations with the HR of 0.87 (Table 2). Age, smoking, alcohol drinking, higher BMI and higher hsCRP concentrations increased the HR for death (Table 2). Regarding prostate cancer, the adjusted CRR indicated statistically significant associations with 25(OH)D and smoking. Higher 25(OH)D concentrations increased the hazard of prostate cancer with the HR of 1.20 but smoking reduced it (Table 2). This somewhat unexpected association between smoking and prostate cancer most probably relates to the association between smoking and 25(OH)D concentrations. In the KIHD cohort, the mean circulating 25(OH)D concentrations are lower among smokers (*n* = 797) compared with non‐smokers (*n* = 1781): ‘summer’ levels 54 vs. 60 nmol l^−1^ (*p* < 0.001) and ‘winter’ levels 37 vs. 39 nmol l^−1^ (*p* = 0.001). In the discussion section, we debate these associations in detail.

The results based on the 25(OH)D measurements at the 11‐year follow‐up visit corresponded with the results based on the baseline measurements. Concerning death, the CRR resulted in the following HRs (95% CI) for the 25(OH)D tertiles, first to third: 1.34 (1.07−1.68, *p* = 0.012), 1.03 (0.81−1.31, *p* = 0.810) and 0.71 (0.56−0.91, *p* = 0.007). The probability of death, excluding prostate cancer death, was 0.47 in the 1st tertile, 0.42 in the 2nd tertile and 0.40 in the 3rd tertile. Regarding prostate cancer, the CRR resulted in the following respective HRs (95% CI): 0.45 (0.27−0.76, *p* = 0.003), 0.93 (0.60−1.43, *p* = 0.720) and 2.03 (1.36−3.03, *p *< 0.001). The probability of prostate cancer was 0.07 in the 1st 25(OH)D tertile, 0.11 in the 2nd tertile and 0.17 in the 3rd tertile. The correlation between baseline and follow‐up 25(OH)D concentrations was 0.274 (*p* < 0.001).

## 4 DISCUSSION

The present competing risk analysis adjusted for 10 covariates together with 25(OH)D evidently showed that higher 25(OH)D concentrations statistically significantly increased the hazard of prostate cancer incidence. In the KIHD cohort, ‘high’ serum 25(OH)D concentrations refer to a mean value of 78 nmol l^−1^ measured in summer and 58 nmol l^−1^ measured in winter that are not high as such but rather within the adequate range (NIH, 2021; Rosen, 2011), which proposes that the direct relationship between circulating 25(OH)D concentrations and the risk of prostate cancer incidence may exist also at desirable 25(OH)D levels. Our results agree with results by Wong et al. (2014), who studied a cohort of 4208 men aged 70−88 years on average for seven years and applied competing risks models.

Broadly, our findings also agree with a meta‐analysis of 21 publications reporting an increased risk of prostate cancer associated with high circulating 25(OH)D concentrations (Xu et al., 2014). However, Xu et al. (2014) together with a longitudinal study by Tuohimaa et al. (2004) suggested a U‐shaped relationship between the risk of prostate cancer and 25(OH)D levels, but our study propose a linear or an inverted U‐shaped relationship, as the absolute risk of prostate cancer in the KIHD cohort peaked at 70 nmol l^−1^ measured in summer and 50 nmol l^−1^ measured in winter (Figure S1). Unfortunately, we are unable to verify this dose–response because the serum 25(OH)D concentrations among the KIHD study participants are low per se but, in any case, our dose–response curve resembles the curve by Wong et al. (2014). Moreover, Xu et al. (2014) speculated the possibility that high 25(OH)D concentrations may relate specifically to aggressive prostate cancer, but we found no difference in the risk of fatal prostate cancer across 25(OH)D tertiles. In general, most malignant prostate tumours are localized, approximately 80% of all cases, followed by regional‐ and distant‐stage diseases (Li et al., 2018), and higher Gleason grade and tumour stage relate to vitamin D deficiency, not high 25(OH)D concentrations (Murphy et al., 2014). Yet another difference between our findings and results reported by Xu et al. (2014) concerns seasons. Xu et al. (2014) observed the relationship between prostate cancer and 25(OH)D concentrations only in individuals with serum collected during summer or autumn, whereas in our study the season did not affect the results.

On the contrary, recent Mendelian randomization studies and a large randomized, 5‐year placebo‐controlled trial testing a 50 μg daily supplementation of vitamin D_3_ found no impact of vitamin D on prostate cancer risk (Jiang et al., 2019; Manson et al., 2020; Ong et al., 2021). These results, all in all, suggest a possible non‐causal relationship between the circulating 25(OH)D concentration and the risk of prostate cancer incidence.

Studies of older individuals per se should apply a competing risk approach instead of the more conventional Kaplan–Maier survival analyses and Cox proportional hazards regressions (Berry et al., 2010). Nevertheless, prospective general population studies of prostate cancer very seldom consider competing risks and the main reason for this probably relates to relatively short follow‐up times. As the interval between 25(OH)D measurements and the follow‐up period, seemingly, effects on the relative risk of prostate cancer incidence specifically when the follow‐up time exceeds 20 years (Grant, 2011), the effect does not concern most prospective studies of prostate cancer. In general, the relative risk of prostate cancer incidence is highest in prospective studies with the shortest follow‐up times and decreases together with increasing follow‐up times (Grant, 2011). From the viewpoint of our study, this may denote underestimated HRs for the risk of prostate cancer incidence.

The few prospective studies of prostate cancer that consider a competing risk approach deal with the metabolic syndrome (Grundmark et al., 2010; Häggström et al., 2014). By and large, associations across prostate cancer, the metabolic syndrome and circulating 25(OH)D levels are complex, as 25(OH)D concentrations appear to modify the relationship between prostate cancer and metabolic syndrome so that the latter increases the likelihood of the former but only when 25(OH)D concentrations are low (Tuohimaa et al., 2007). Among the KIHD study participants, most of whom have insufficient serum 25(OH)D levels, the metabolic syndrome increases the risk of prostate cancer (Laukkanen et al., 2004).

In addition to the relationship between prostate cancer and metabolic syndrome, levels of circulating 25(OH)D appear to complicate the relationship between prostate cancer and smoking. Overall, smoking increases the risk of prostate cancer (Huncharek et al., 2010). In the present study, however, smoking is associated with a lower risk of prostate cancer, and we suggest this result is due to the inverse relationship between circulating 25(OH)D concentrations and smoking. In other words, as smoking associates with reduced circulating 25(OH)D levels (Jiang et al., 2016; Kassi et al., 2015; Virtanen et al., 2011), and if lower 25(OH)D concentrations are associated with the reduced risk of prostate cancer incidence, it is possible to detect a negative correlation between smoking and the risk of prostate incidence without causality.

### 4.1 Strengths and limitations

The main strength of this study is its good reliability. Owing to the KIHD 25(OH)D measurements at the follow‐up visit, we were able to repeat our analyses and compare their results to the results based on baseline measurements.

The main limitations of this study relate to a lack of genetic information. Prostate cancer is a heritable disease, and an individual's genotype, such as vitamin D receptor polymorphisms (Li et al., 2007), modulates its association with the vitamin D status. In addition, the comparatively small sample size together with the incompleteness of data did not allow us to investigate the relationship of 25(OH)D with different tumour stages. Rectal examinations and prostate‐specific antigen tests at baseline would have improved the reliability of our inclusion criterion, that is, no evidence of disease.

## 5 CONCLUSION

Our findings proposed a direct relationship between circulating 25(OH)D concentrations and the risk of prostate cancer incidence. The findings did not support the hypothesized explanation for this relationship that longer life expectancy due to higher 25(OH)D levels leads to a higher probability of prostate cancer incidence. The competing risk analysis did not eliminate the relationship between 25(OH)D and prostate cancer incidence but rather strengthened it. Prospective studies of prostate cancer should consider a competing risk approach, specifically, when the follow‐up time exceeds 20 years.

## CONFLICT OF INTEREST

Authors have no conflict of interest.

## Supporting information

Fig S1

## Data Availability

The data that support the findings of this study are available from the Institute of Public Health and Clinical Nutrition, University of Eastern Finland, upon reasonable request.
