# Supplementary figures and images for: How competing risks affect the epidemiological relationship between vitamin D and prostate cancer incidence? A population‐based study
Source: Andrologia. 2022 Feb 28;54(6):e14410. doi: 10.1111/and.14410 (PMC9540471; doi:10.1111/and.14410)

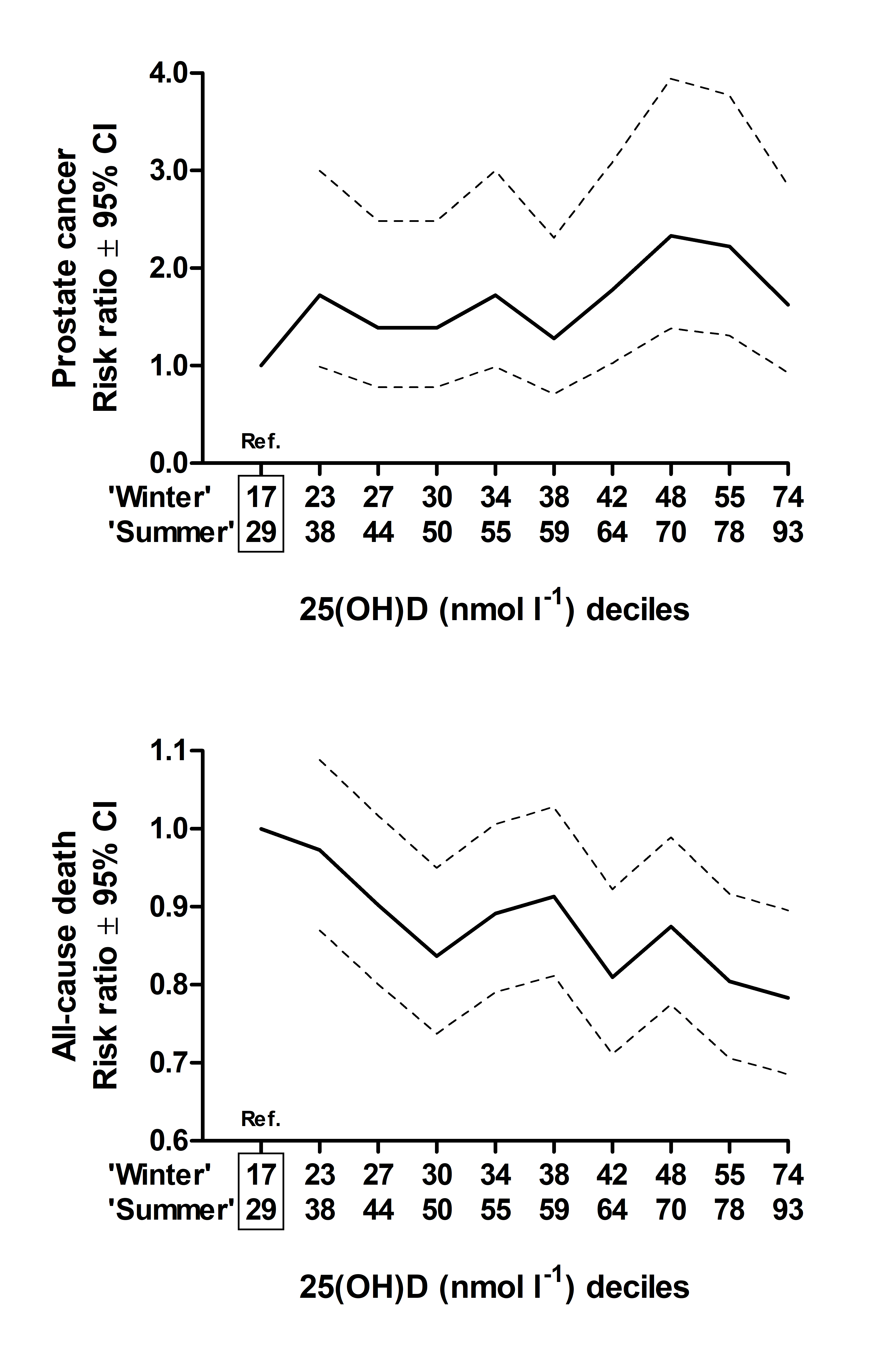

Supplement: Supplementary file 1 — Fig S1 [file AND-54-e14410-s001.jpg]
